# Magneto-Impedance Biosensor Sensitivity: Effect and Enhancement

**DOI:** 10.3390/s20185213

**Published:** 2020-09-12

**Authors:** Abkar Sayad, Efstratios Skafidas, Patrick Kwan

**Affiliations:** 1 Department of Neuroscience, The Alfred Centre, Central Clinical School, Monash University, Melbourne, Victoria 3004, Australia; abkar.sayad@monash.edu; 2 Department of Electrical and Electronic Engineering, Melbourne School of Engineering, The University of Melbourne, Victoria 3010, Australia; sskaf@unimelb.edu.au

**Keywords:** magneto-impedance, impedance, magnetic materials, magnetic particles, biosensor, sensitivity, magnetic field

## Abstract

Biosensors based on magneto-impedance (MI) effect are powerful tools for biomedical applications as they are highly sensitive, stable, exhibit fast response, small in size, and have low hysteresis and power consumption. However, the performance of these biosensors is influenced by a variety of factors, including the design, geometry, materials and fabrication procedures. Other less appreciated factors influencing the MI effect include measuring circuit implementation, the material used for construction, geometry of the thin film sensing element, and patterning shapes compatible with the interface microelectronic circuitry. The type magnetic (ferrofluid, Dynabeads, and nanoparticles) and size of the particles, the magnetic particle concentration, magnetic field strength and stray magnetic fields can also affect the sensor sensitivity. Based on these considerations it is proposed that ideal MI biosensor sensitivity could be achieved when the sensor is constructed in sandwich thick magnetic layers with large sensing area in a meander shape, measured with circuitry that provides the lowest possible external inductance at high frequencies, enclosed by a protective layer between magnetic particles and sensing element, and perpendicularly magnetized when detecting high-concentration of magnetic particles.

## 1. Introduction

There is increasing interest in using high-performance biosensors for biomedical applications. Among them, biosensors based on magneto-impedance (MI) effect are widely used in medical diagnosis as they exhibit high sensitivity and fast response. However, the performance of these biosensors is influenced by a variety of factors. In this article, we review advances in thin films MI effect sensors and consider how the biosensor sensitivity varies based on factors such as design, geometry, materials and fabrication procedures. We review less appreciated but important concepts such as how measuring circuit implementation influences the MI effect, and how the sensitivity varies with the material used for construction and the geometry of the thin film sensing element. Sensor geometries, compatible with the interface microelectronic circuitry, are compared to wires and ribbons. We also review the relationship between MI effect sensitivity and the type and size of particles, including magnetic, ferrofluid, beads, and nanoparticles. Lastly, the effects of liquid, magnetic particle concentration, magnetic field strength and stray magnetic fields on biosensor sensitivity, with specific focus to MI effect, are considered.

### Magneto-Impedance Effect

MI effect refers to the change of the electrical impedance in magnetic materials when exposed to an external magnetic field. It was introduced in the 1990s to detect changes of impedance in amorphous wires, due to changes in skin effect losses [1,2]. The MI effect first was described by Rayleigh et al. [3] and further investigated by Kittel et al. [4]. It is associated with skin effect, which is the penetration of the electromagnetic field within a conductive material or the region near the surface of a conductor where alternating current (AC) flows through the material. The amplitude of the electromagnetic field decrease from the conductor surface is defined by the penetration depth δ and it is expressed by Equation (1):

(1) $$\delta =\frac{1}{\sqrt{\pi f\sigma \mu }}$$

where *f* describes the AC frequency, *σ* and *μ* denote magnetic material conductivity and permeability, respectively. In practice, the penetration depth δ determines the specimen/sample’s efficient cross-section when AC flows through it. If a sample possesses magnetic properties and is subjected to an external magnetic field, the employed field modifies its permeability resulting in a MI effect. In short, the fundamentals of MI effect are determined by Maxwell equations and further described in articles on electromagnetism [5,6].

**Figure 1:**
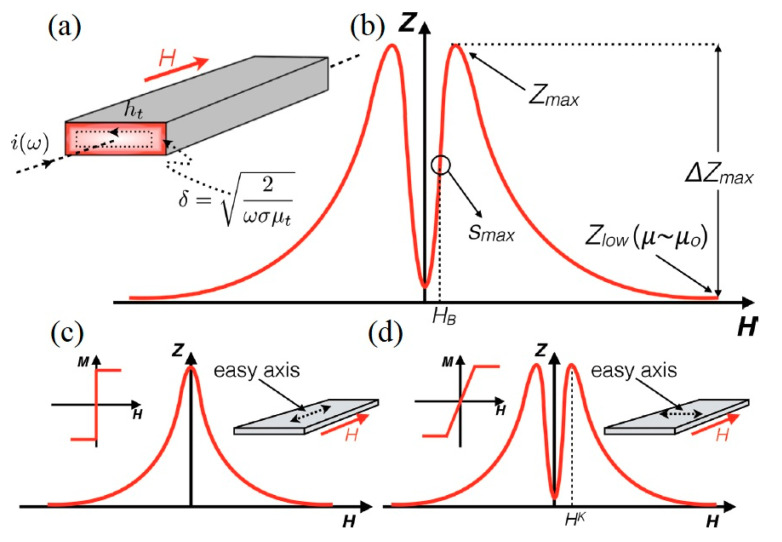
Magnetization process configuration. (**a**) Planar sample showing MI effect. (**b**) MI curve. (**c**) Longitudinal anisotropy displayed by single-peaked MI curve. (**d**) Transverse anisotropy displayed by double-peaked MI curve. Reprinted with permission from [9], under the Creative Commons Attribution (CC BY 3.0) license.

When the sample structure is uncomplicated, Maxwell’s equations can be analytically solved to derive the resulting impedance. Examples of simple structures include a uniform thickness sheet 2*a* and unlimited dimensions. In this case the impedance can be determined using Equation (2) [7]:

(2) Z=Rdc√jθ coth √jθ

where *R_dc_* denotes the direct current resistance, j = √−1, and *θ* = *a* √2*πfσμ* = √2*a*/*δ*. Equations for the impedance of wires are also possible and can be expressed in terms of Bessel functions [7]. As illustrated in Figure 1, the variation in permeability in relation to an alternating magnetic field generated by a flowing current can be substituted for the values of permeability in Equations (1) and (2). 

The MI effect of a magnetic material is usually expressed as the relative change of impedance when an AC flows in a conductor that is submitted to a magnetic field. It is given by Equation (3), and is expressed in terms of *Z_min_,* which is the minimum measured impedance when the magnetic material resides in a magnetic field:

(3) $$MI\left(\%\right)=\frac{Z-Z\min}{Z\min}\times 100$$

In practice, *Z_min_* is the minimum impedance when the magnetic field strength magnetically saturates the material. The impedance can be also measured when magnetic field is off i.e., when *Z_min_ = Z (H = 0).* As shown in Figure 1b, *Z_max_* represents the peak value of the MI whilst *Z_low_* denotes the lowest value of the MI. The maximum for MI is achieved when the value of *Z* is at its maximum. This occurs at the field intensity H where the permeability of the sample is maximum, and the skin depth is minimum. Judicious choice of magnetic field is required to achieve the largest MI_max_. 

A critical consideration in sensor design is sensitivity, *S_max_*, and its relationship to MI ratio. The maximum sensitivity occurs when *Z*(*H*) curve exhibits a maximum change when magnetic field is applied as shown in Figure 1. The *Z(H)* curve is influenced by anisotropy where longitudinal anisotropy occurs when the magnetized sample is orientated parallel to the applied field and the current flow as shown in Figure 1c. Whilst, transverse anisotropy occurs when the magnetized sample is orientated perpendicular to the applied field and the current flow as shown in Figure 1d. For the longitudinal anisotropy configuration, at *H* = 0, the transverse permeability is maximum and indicated by *Z*(*H*) curve that poses a single peak. For transverse anisotropy, the transverse permeability is maximum at H^k^, given by Equation (4) [8,9]:

(4) $$H^{k}=\frac{2K}{\mu_{0}M_{s}}$$

where *K* denotes the anisotropy constant and *M_s_* describes saturation magnetization. The maximum sensitivity is achieved when MI is maximum and H^k^ is at its minimum. For maximum sensitivity it is crucial to achieve a sharp transverse magnetic anisotropy for thin films biosensor applications.

MI sensor sensitivity response can be enhanced with the aid of magnetic microparticles or superparamagnetic nanoparticles who exhibit varying magnetic, physical and chemical properties. These particles vary in size from less than 100 μm for magnetic particles and less than 100 nm for nanoparticles [10,11,12,13,14,15,16,17]. These particles are magnetized when subjected to a magnetic field and demagnetized when the magnetic field is removed and can be used to detect targets such as protein, cells, enzymes and deoxyribonucleic acid. An additional feature of these particles is that they can be functionalized to bind and capture molecular targets of interest to assist with the detection process. Magnetic particles exhibit physicochemical stability enabling them to detect the targets with no deleterious biological effects and importantly do not chemically interact with the target that is sought. Consequently, they have been extensively utilized in medical applications such as immunoassays [18], magnetic resonance imaging (MRI) [19], nucleic acid detection [20] and magnetic separation [21]. 

**Figure 2:**
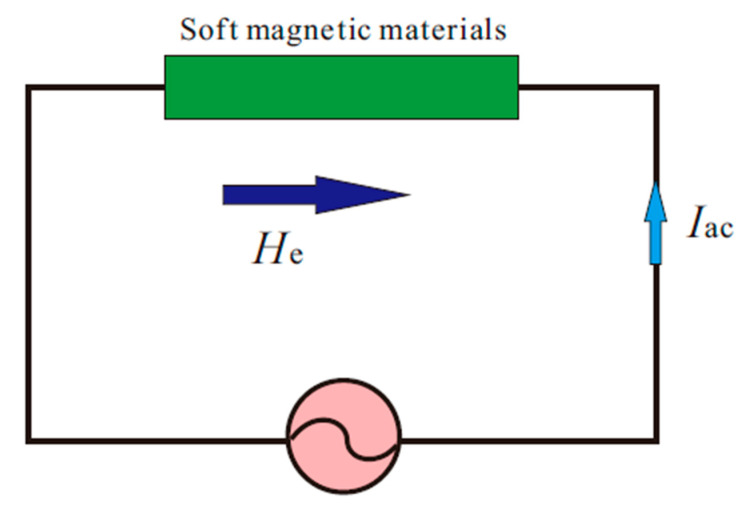
MI effect in soft magnetic materials. Reproduced with permission from Elsevier [49].

After the introduction of magnetic biosensors by Baselt et al. [22] in 1998, magnetic particle based magneto-resistive sensor principles have been extended to resonance sensors [23,24,25,26], fluxgate sensors [27,28], hall effect sensors [29,30,31,32], magneto-resistance sensors [33,34,35,36,37,38,39], spin valve sensors [40,41,42,43], and anisotropic magneto-resistance sensors [44,45,46]. However, MI effect sensors have received great attention in sensing of biological magnetic fields. The MI effect is expressed as the relative change of the impedance of the soft magnetic materials when an AC flows through them when an external magnetic field “*He*” is applied, as shown in Figure 2 [1,2,47,48]. 

Similarly, the sensor sensitivity can be described by magneto-resistance (MR) and magneto-reactance (MX) effects. They are expressed as the relative change of resistance and reactance when an AC flows through the soft magnetic material in the presence of an external magnetic field and given by Equations (5) and (6):

(5) $$MR\left(\%\right)=\frac{R\left(H\right)-R\left(H\max\right)}{R\left(H\max\right)}\times 100$$

(6) $$MX\left(\%\right)=\frac{X\left(H\right)-X\left(H\max\right)}{X\left(H\max\right)}\times 100$$

## 2. Factors Influencing the Magneto-Impedance Effect

The geometry, materials and structure of the sensors are major factors that influence the MI effect. Table 1 summarizes the MI ratios achieved by different constructed sensor geometry, materials, and structure.

### 2.1. Sensor Geometry

Biosensor geometry plays a critical role on the sensor’s MI response. Amorphous wires and glass-coated materials with MI effect of 600% have been reported [50,64] when geometry and magnetic domains were aligned in the transverse direction (transverse permeability) [50,65]. Results for amorphous ribbons were presented in [66,67]. An important consideration in biosensor design is the magnetic material thickness. Thicknesses of tens of microns for both wires and ribbons are sufficient to achieve high MI response at low frequency, 10–100 kHz when the skin depth matches the material thickness whereas at higher frequencies of 1 GHz, thicknesses of 1 µm thick are required. 

**Figure 3:**
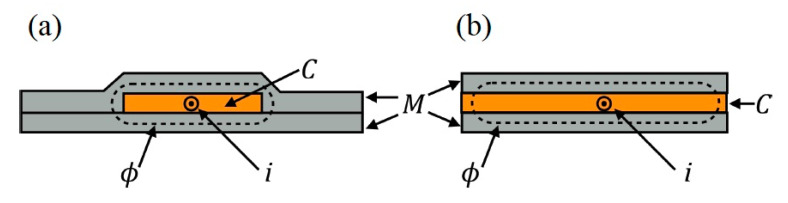
Sandwich structure MI biosensor (*M* denotes magnetic layer and C refers to conductive). (**a**) Closed path magnetic flux *ϕ* as conductive layer is enclosed by the magnetic layers. (**b**) Open path magnetic flux *ϕ* as conductor is not enclosed by magnetic layers resulting in low MI effect. Reprinted with permission from [9], under the Creative Commons Attribution (CC BY 3.0) license.

Sandwich structures, comprising of a double magnetic layer, deposited on either side of a conductive layer (non-magnetic) has shown to enhance MI in thin films [52,53,54,56,57,68]. This enhanced MI effect in sandwich structures has been attributed to the large permeability magneto-inductive effect whilst allowing it to overcome the skin effect that usually results in large impedance variations. The largest MI ratio for sandwich structure biosensors, where wire-shaped sample was employed, has been 800% as shown in Table 1 [51]. Two potential configurations are possible to construct sandwich structure and are illustrated in Figure 3. Figure 3a resembles the shape of a core shielded wire where the conductive layer is completely enclosed by the outer magnetic layers. In this layout, the magnetic flux produced by the AC faces a closed path resulting in high permeability. The configuration shown in Figure 3b, magnetic and conductive layers have the same width, the magnetic open-flux created by the AC field crosses a low permeability path diminishing the resultant MI effect [9,69].

The open-flux sandwich configuration is easily fabricated in one step as compared to the complex fabrication required for the closed-flux configuration. Closed-flux configuration requires large material thickness resulting in high MI response [70]. 

The sandwich structures are usually patterned as rectangular stripes, meanders, or ellipsoids. These patterns are adjustable to match their lateral dimensions to increase the MI effect. Meander line patterns can be employed to further enhance the MI effect in Permalloy and ferromagnetic materials [71]. Thick magnetic material exhibits low permeability achieving high MI response in closed-flux configuration sandwich structure compared to open-flux layout. Meander line pattern is the best adjustable shape to provide large MI response. 

### 2.2. Sensor Materials

Magnetic material selection is a crucial parameter to improve sensor sensitivity. MI biosensor’s most widely used and successful materials are amorphous alloy, nickel-cobalt-silicon-boron (Fe-Co-Si-B or similar), made by rapid melt quenching. MI effect in the vicinity of 700% has been reported for magnetic material made of amorphous alloy CoSiB in sandwich structure with closed-flux configuration [52]. Such extraordinary performance was obtained when CoSiB sandwich sensor structure (2µm thick) was enclosed by an insulating layer silicon dioxide (SOi_2_) between the conductor copper (Cu) and outer magnetic layer (CoSiB) which prevented AC current from penetrating the magnetic layers. Theoretically, the MI ratio is predicted to be between 400−500% at frequencies of 10−100 MHz for sandwich structure of amorphous alloy films (CoSiB) or Permalloy Nickle-Iron (NiFe) with 1μm thick middle conductive layer. In practice, when the thickness is reduced to 0.1 μm, the MI ratio falls significantly to 15–20% of its peak value at frequencies of 300–400 MHz [72]. MI ratios of; 5.4% in amorphous alloy FeSiB with thickness of 2.7 μm [58], 100% in 2 μm thick ferromagnetic material cobalt-niobium-zirconium (Co-Nb-Zr) [59], and 205% in nanocrystalline Fe-Cu-Nb-Si-B with thickness of 10 nm and width of 1 mm films have been reported [73]. 

Recently, Permalloy magnetic material with the composition of 20% of Iron and 80% of nickel (Fe_20_Ni_80_) has received significant interest in the construction of MI biosensors. This is because Permalloy exhibits large permeability, low magnetostriction and low crystalline anisotropy. Permalloy is a very soft magnetic material; abundant, low cost and extensively used as a base material to enhance anisotropic magneto-resistance (AMR) sensors and MI biosensors [74]. Various thicknesses of Permalloy thin films have been made using vapor deposition techniques. Thinner films usually exhibit a high MI response. However, increasing thickness has advantages including an easier to define magnetization axis, that is vertical to the sample plane, and the material does not enter the transcritical state [75]. As has been previously discussed, increasing thickness of amorphous materials diminishes the magnetic softness of the material and consequently diminishes the MI performance. Ideally to maximize the MI effect requires both careful consideration and design of magnetic softness and layers thickness [76]. Optimal thickness of 170 nm has been reported when thin films were prepared by low pressure sputtering deposition [77]. To create thicker magnetic layers, that are below the critical thickness, thin spacers between multilayers such as titanium, copper, silver, etc., have been used [78,79]. High MI effect has also been achieved in sandwich layout of amorphous ribbon and wires or Permalloy materials when the outer magnetic layer is below the critical thickness. 

### 2.3. Sensor Fabrication Structures

Thin film MI biosensors are commonly fabricated by techniques such as physical vapor deposition [80,81,82], electrodeposition [61,83,84,85] or flame plating [86]. Fabrication methods contribute greatly to MI performance variation. Sputtering is a favorable option as compared to evaporation methods as its parameters are dependent on the tool used and the material selection. Precise fabrication procedures and equipment provide superiors MI biosensors. Parameters such as the substrates, tools, materials, procedures, and recipes are of utmost importance in sensor fabrication. For example, glass, silicon, and polymer substrates are widely used in MI biosensor fabrication [56,87,88,89,90]. High sensor sensitivity is usually achieved by defining the magnetic domain during film deposition by applying an external magnetic field regardless of the material type. However, it is rare to have a built-in magnetic field source and heating block in a magnetic material deposition tool to aid in defining the transverse anisotropy. Therefore, magnetic domain is sometimes defined by annealing after deposition. Alternatively, permanent magnets can be used to define the magnetic domain during the electrodeposition of thin films, limiting the use of the annealing process [61].

Fabrication parameters should be adjustable to the measurement circuits to achieve the required MI ratio. For example, substrates can be diced into the preferred shape to fit in the measurement setup. Moreover, the patterning should be simple and modifiable to fit any measuring circuits to maximize the MI response. Photolithography is used to create shapes with exceptional lateral resolution and very distinct borders [91]. Photolithography is used to pattern thin films that are then built-up on the pattern using techniques such as deposition, etching and lift-off [92]. Photolithography is one the simplest method of MI biosensors patterning. Meander line shapes constructed using photolithography, with transverse anisotropy defined during deposition, exhibit a higher MI effect (190%) [62], compared with those of the same shape (183%) without transverse anisotropy defined during deposition [61]. Therefore, the fabrication process can be optimized to enhance the MI response; for example, the Permalloy multilayer structure, choice of open- or closed-flux configuration [70], magnetic layer thickness [93], magnetic layer spacers [94] and variation in conductive layer thickness [95].

The MI effect depends considerably on the lateral dimensions of the sensor pattern, where improper downscaling alters its magnetic properties. The transverse anisotropy is altered in elongated-shape MI biosensors which impacts the sensor’s sensitivity [96]. In this case, ellipsoid lateral dimension adjustment is required to correct transverse anisotropy [63]. Another important issue is flux leakage which increases in an open-flux multilayer layout sensor as the sensor’s lateral size decreases [97]. This is because the induced domain is changed, which worsens the MI effect. The highest MI response has been reported for a circular-shaped wire made of FeCoNi magnetic tubes in a sandwich structure (Table 1). 

## 3. Measurement Systems Influencing the Magneto-Impedance Effect

### 3.1. Measurement at High Frequency Influencing MI Effect

To achieve a greater MI response, the operational frequency should be in the range of 10–100 MHz. However, to achieve this level of MI effect required an accurate experimental setup and mathematical analysis of the obtained data and impedance values [98]. At low frequencies, the current and voltage are constant for every point of the circuit at any given moment in time. However, at high frequencies, when the electromagnetic wavelength signal *λ* is equivalent to the measuring circuit length expressed by Equation (7), these values vary across the circuit at any instant in time. Hence, the measured impedance oscillates between the minimum and maximum values in relation to the operational frequency:

(7) $$\lambda =\frac{v}{f}$$

**Figure 4:**
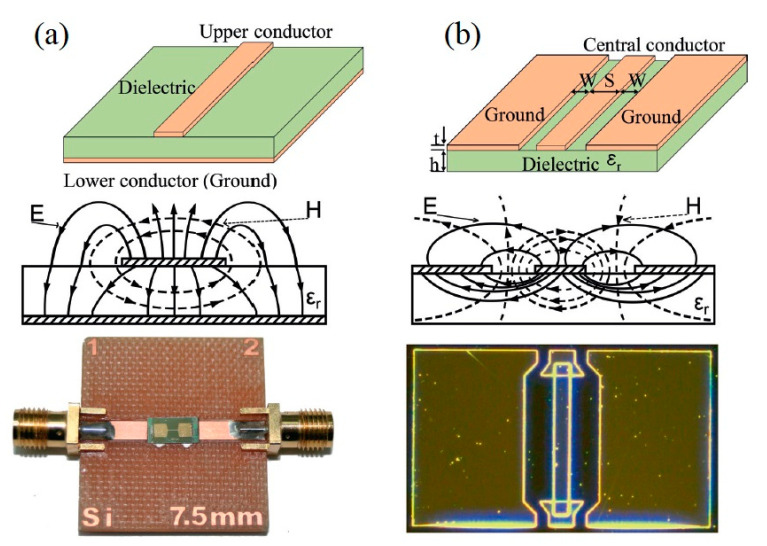
MI measurement circuits based on transmission lines. (**a**) Microstrip line where conductor is at the top and ground at bottom. (**b**) Coplanar line where the conductor and ground are on the top surface. Reprinted with permission from [9], under the Creative Commons Attribution (CC BY 3.0) license.

Here *λ* is the electromagnetic wavelength signal, *v* is the velocity of light and *f* represents the signal frequency. To measure the impedance at high frequencies, a network/impedance analyzer or specialized impedance-measuring systems is required. Therefore, for accurate measurement of the impedance of these circuits a device holder and matched impedance transmission lines such as waveguides and coaxial cables are desired. As examples, microstrips and coplanar waveguides shown in Figure 4 can be used in planar devices. 

Coplanar waveguides comprise of a dielectric substrate that has both ground and signal conductors on the top face of the substrate in a configuration shown in Figure 4b. Both the device and the waveguides are produced by lithography and can be characterized using a probe station [99]. Microstrip configuration is shown in Figure 4a, where the device is placed between two microstrip lines that have external radio frequency connectors. This is an effective setup for achieving a high MI effect, however calibration is required to determine and account for the parasitic resistance and conductance in the coaxial cable [98].

### 3.2. Measuring Circuit Influencing MI Effect As a Function of Frequency

**Figure 5:**
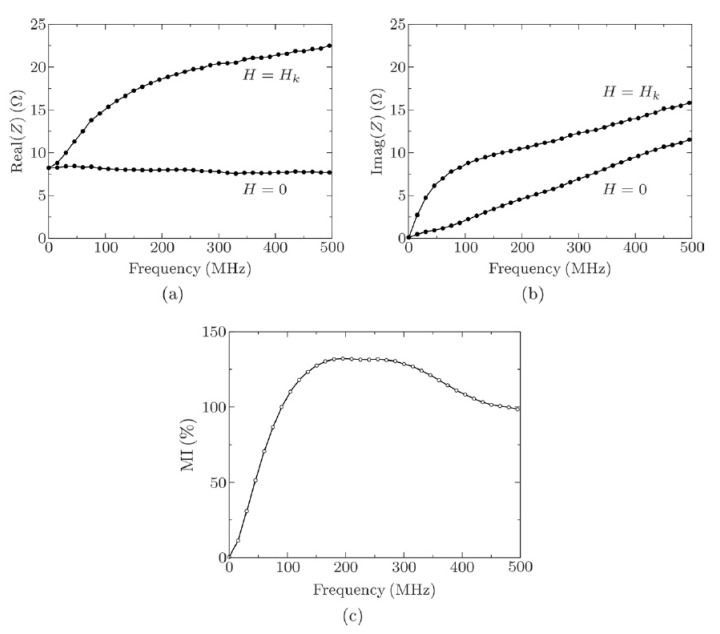
Impedance results measured as a function of frequency. (**a**) Impedance real part. (**b**) Impedance imaginary part. (**c**) Magneto-impedance ratio. Reprinted with permission from [9], under the Creative Commons Attribution (CC BY 3.0) license.

The MI effect is typically measured when the sensor is excited by a magnetic field that is generated by Helmholtz coils or permanent magnets. Impedance has in phase and a 90 degree out of phase component, denoted as real and imaginary components, as is typically measured by network or impedance analyzers, especially for high frequencies. It is measured over a frequency and provides details of MI effect variation in the frequency domain through its real and imaginary parts. Figure 5 represents the measured impedance of thin film NiFe/Au/NiFe with a defined transverse anisotropy. 

The impedance’s imaginary and real parts are plotted at *H* = 0 and *H* = *H_k_*. The impedance component are plotted when transverse permeability is equivalent to *µ0* and maximum [100]. It is worth noting that in a sandwich structure, the magneto-induction effect dominates over the skin effect. Figure 5a indicates that the real part of the impedance at *H* = 0 is constant relative to the sample resistance because the penetration depth *µ0* is greater than the NiFe/Au/NiFe film thickness. In contrast, when *H* = *H_k_*, the real impedance part increases as the penetration depth is lesser than the NiFe/Au/NiFe film thickness. Thus, the impedance real part increases with the frequency. At *H* = 0, the impedance imaginary part increases linearly from *f* = 0, as the slope relates to the measuring circuit inductance and the sample [98]. On the contrary, when *H = H_k_*, at frequencies less than 100 MHz, the imaginary part increases rapidly, resulting in a high impedance change ratio. Therefore, the relative change in the imaginary part is reduced as the frequency decreases. The MI response completely depends on the real and imaginary impedance parts values. It increases with frequency, as indicated by the values of the real part. Whereas the imaginary part exhibits low values because of the external inductance caused by the measuring circuit. In fact, to maximize the MI effect as a function of frequency real and imaginary impedance parts must be kept approximately equal. To maximize the values of the imaginary part, the external inductance must be reduced. 

Therefore, it is critical to employ a measuring system that has the lowest possible external inductance. Transmission lines (microstrip and coplanar) should be used, taking into consideration the external inductance they generate when measuring the MI effect. A specialized technique, (de-embedding), is needed to obtain the essential impedance of the sample [99,101]. This allows for a comparison of the measured MI values with theoretical predictions.

## 4. Magnetic Particles Influencing the Magneto-Impedance Effect 

The type and size of magnetic ferrofluid, Dynabeads, and nanoparticles can also affect the sensor sensitivity. Table 2 illustrates the highest MI ratio based on different types of magnetic particle, where a large MI response was achieved when sensors incorporated ferrofluid magnetic particles.

### 4.1. Magnetic Ferrofluid Detection

**Figure 6:**
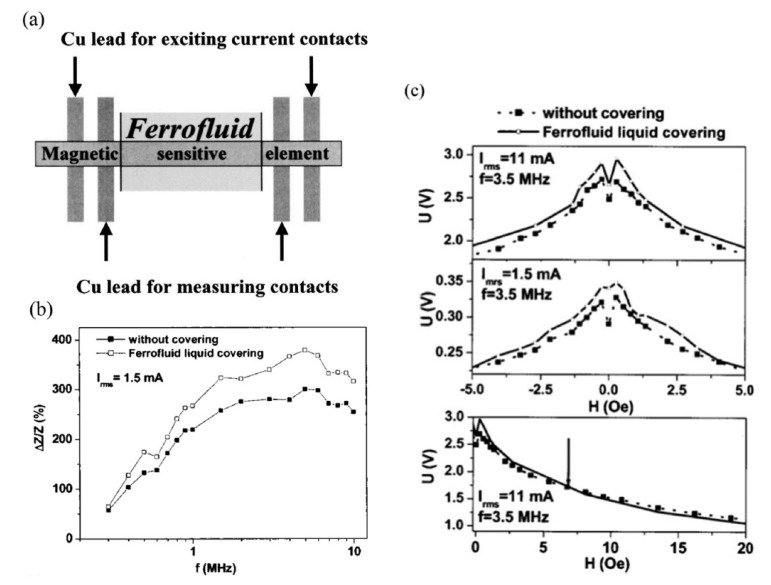
Detection of magnetic ferrofluid. (**a**) MI biosensor schematic using test setup. (**b**) MI effect based on Frequency. (**c**) MI effect based on magnetic field when covering the sensing element and when uncovering. Reprinted from [102], with the permission of AIP Publishing.

Magnetic ferrofluid detection was first introduced in 2003 when it was incorporated in a commercial prototype MI biosensor based on a Vitrovac CoFeMoSiB ribbon [102]. The sensor’s sensitivity was investigated based on the magnetic ferrofluid, magnetic field value, and driving current. As shown in Figure 6a, the four-points method was employed to measure the MI effect. Two points were used to excite the current contacts, while another two points were Cu-leads utilized for measuring conductivity. Ferrofluid magnetic particles of 10 nm size were used and spread consistently over the sensing element of the biosensor. Helmholtz coils parallel to the sensing element axis were used to generate a magnetic field “*He*” of ±150 Oe and, the driving current was applied in the longitudinal direction of the sensing element (0.3–10 MHz). Ferrofluid magnetic beads were magnetized as they were subjected to the external magnetic field, “*He*”, and the AC. The beads generated a stray magnetic field that modified the superimposed magnetic field, thereby altering the MI effect.

As shown in Figure 6b, the MI effect is significantly enhanced at frequencies (1–10 MHz) as ferrofluid beads are located on the sensing element (beads in direct-contact with the sensing element). Furthermore, sensor sensitivity depends on whether the testing technique is direct-contact or non-direct contact. Such that, the non-direct contact method is safer for detecting magnetic particles unlike direct-contact method where biochemical liquids contaminate the sensor’s sensing element. In addition, Kurlyandskaya et al. reported that the MI effect is greatest at magnetic fields less than 6 Oe and reduces at higher values in the presence of the magnetic ferrofluid, as shown in Figure 6c [102]. However, theories describing this phenomenon are not well developed. A later study detected a ferrofluid containing Iron oxide nanoparticles and spherical Iron particles (500 nm) using a MI biosensor made from Permalloy materials [103]. Yuvchenko et al. stated that the number of Iron particles is proportional to the strength of the stray magnetic field [103]. The more particles, the stronger the stray magnetic field and the greater the MI effect. However, this relationship is invalid if a cluster of beads or agglomeration is created when the concentration is too high. They also reported that an increase beyond 4% ferrofluid concentration triggers a decline in the MI effect. This drop was attributed to the presence of residue on the sensing element of the biosensor meander line, which is inconsistent with the findings of Kurlyandskaya et al. [102].

Recently, Kurlyandskaya et al. linked the drop in the MI effect to the presence of magnetic ferrofluid particles and a high-field hysteresis rise when testing on an electroplated CuBe/FeCoNi wire sensor [104]. However, Amirabadizadeh et al. negated these findings by reporting a greatly improved MI effect tested on a CoFeSiB amorphous wire biosensor using magnetite ferrofluid [105]. They stated that, in the presence of ferrofluid, the maximum MI ratio increased from 315% to 417% and the sensitivity increased approximately 150% at frequency of 4.5 MHz. These findings are not entirely congruent with the MI effect values reported by Kurlyandskaya et al., who observed an MI ratio decrease from 108% to 104% in the presence of ferrofluid particles [102,104]. The disparity in these results is not certain and may be due to differences in the two testing methods used and in the superimposed magnetic fields induced by different ferrofluid distributions. However, it is worth noting that sensor sensitivity is enhanced at high-concentration magnetic ferrofluid beads regardless of size as no cluster or agglomeration of beads is formed.

### 4.2. Magnetic Dynabeads Detection 

#### 4.2.1. Dynabeads M-450 Detection on Ribbon Films

Dynabeads M-450 (4.5 μm) were recently used as a magnetic label for bioassay detection based on the MI effect. Kurlyandskaya et al. tested two samples, a ribbon thin film sensor in a phosphate buffered saline (PBS) containing the Dynabeads and PBS without Dynabeads, measurements were conducted using the four points technique with direct-contact method [107]. The results confirmed a variation in the MI effect in accordance with bead concentration. The observed MI ratios were 300% with and 275% without beads. The maximum observed difference was 25% with this sensitivity observed at a field of ±4 Oe. However, sensor sensitivity can be enhanced at lower beads concentration. 

#### 4.2.2. Dynabeads M-480 Detection on Sandwich Thin Films

Dynabeads M-480 are similar to Dynabeads M-450, and both have been detected using a MI biosensor. Kurlyandskaya et al. observed an enhancement in the MI ratio when detecting Dynabeads M-480 at magnetic fields below 6 Oe, followed by a drop when it exceeded 6 Oe [106]. This result is in accordance with previous finding in which ferrofluid particles were detected [102]. In addition, they observed a curve shift in the MI response due to the presence of a cluster of particles and it is referred as offset MI effect.

It is worth noting that Kurlyandskaya et al. have fabricated a multilayer MI biosensor using ribbon magnetic materials placed between two Au films with Dynabeads M-480 as a magnetic label [106]. The Au films improve the biocompatibility of the MI biosensor. Though, the skin effect of the AC was greatly diminished due to the excellent conductivity of the Au film, as it provides a major path for AC current to flow. An improvement to this sensor may be achieved by placing an insulating layer between the conductor and the magnetic material. 

#### 4.2.3. Dynabeads MyOn Streptavidin C1 and M-280 Streptavidin Detection 

Two sizes of streptavidin, MyOne Streptavidin C1 (1 µm) and M-280 Streptavidin (2.8 µm), were employed as magnetic labels in a Co-based ribbon MI sandwich film biosensor to investigate the MI effect [108]. Yang et al. used an insulating layer (SiO_2_) placed between the amorphous ribbon and the Au film to protect the ribbon from contamination and maintain a high MI response [108]. There was a perceptible difference in the MI ratio when detecting each Dynabead size (1 µm and 2.8 µm), unlike the case with Dynabeads M-480 and M-450. The same amounts of both Dynabeads, sizes (1 µm and 2.8 µm), were detected by the MI biosensor. The MI ratio was about 80% for the 2.8 µm bead size and 70% for the 1 µm bead size. The lowest detectable concentration of Dynabeads was (5 μg mL^−1^) for 1 μm Dynabeads and a 1 μg mL^−1^ concentration for 2.8 µm Dynabeads. It is also worth noting that MI sensor sensitivity is enhanced at lower concentration regardless of the beads size. 

### 4.3. Magnetic Nanoparticles Detection 

#### 4.3.1. Nanomag-D Beads Detection on Ribbon Films

Nanomag-D beads were utilized as a magnetic detection label with a MI biosensor made of amorphous ribbon to investigate sensor sensitivity based on MI, MR, and MX effects [109]. There, the sensor surface was treated by nitric acid to further enhance the MI effect. It was reported that MI effect increased in the acid-treated ribbon compared to untreated ribbon sensor. The increase in sensor sensitivity was attributed to the effect of micro-holes pattern created on the surface of the ribbon by the acid. 

Devkota et al. have also investigated MR and MX effects in addition to MI effect [109]. There it was reported that the MI ratio was lower than the MR and MX ratios, (7%, 16% and 23%), respectively. Devkota et al. presented a new and simple technique based on sensor surface treatment and Nanomag-D beads detection for improving sensor sensitivity [109]. The relative contribution to MI enhancement due to Nanomag-D beads and the nitric acid sensor surface treatment was not established. Further insights might be achieved when experiments are conducted to test surface-treated sensors with various magnetic beads, such as Dynabeads, to further confirm the source of sensor sensitivity enhancement.

#### 4.3.2. Sandwich Thin Films for Detection of Ferrogel

Nanoparticles suspended in ferrogels have been extensively employed in MI biosensing applications. Kurlyandskaya et al. fabricated a MI biosensor based on FeNi/Ti_3_ magnetic materials to detect ferrogels suspended in a stable aqueous solution of γ-Fe_2_O_3_ magnetic nanoparticles [110]. Different concentrations of Iron oxide ferrogel particles were detected and referenced to a blank sample. The results showed an increase in MI ratio compared to a blank sample. The magnetic signal, measured using a network analyzer, was used to derive the MR and MI ratios. A 50% Oe sensitivity was demonstrated in both MR and MI responses, with the field interval being higher in the MI response. The MI response increase is described by the presence of Iron oxide ferrogel particles. This is in compliance with previous findings, which all found higher sensitivity in the presence of magnetic particles, regardless of type [102,107]. 

#### 4.3.3. Iron Oxide (Fe_3_O_4_) Nanoparticles Detection 

Iron oxide nanoparticles are another effective candidate for biosensing using MI biosensors. Devkota et al. has applied Iron oxide (Fe_3_O_4_) nanoparticles on amorphous ribbon biosensors using a non-contact testing method [109]. There, the amorphous ribbon was enclosed by a thin parafilm to prevent direct-contact between nanoparticles and the sensor sensing element [111]. Here, It was observed that the MX biosensor demonstrated greater sensitivity than MR and MI biosensors when detecting low concentrations [111]. It is worth noting that the MI, MX and MR responses remained almost unchanged at concentrations above 124 nm. 

## 5. Magnetic Particle Concentration and Magnetic Field Influencing Magneto-Impedance Effect

### 5.1. Effect of Magnetic Particle Accumulation on MI Response

**Figure 7:**
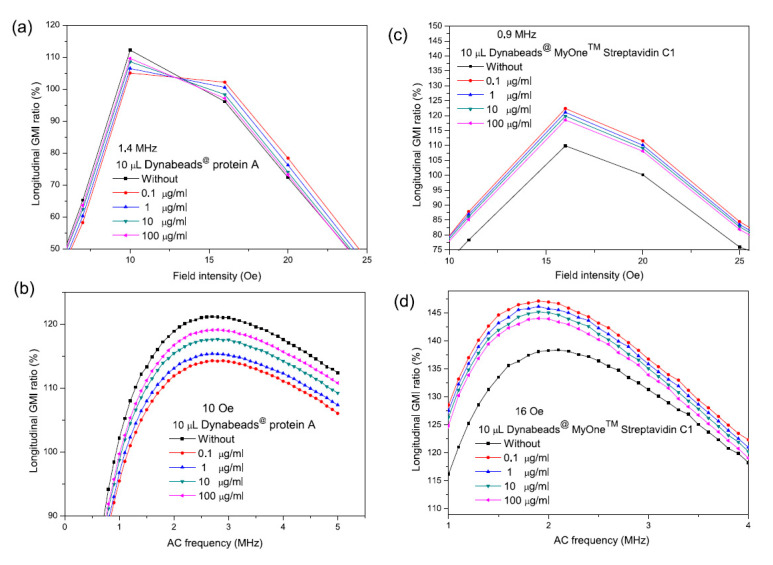
MI response based on magnetic particles concentration. (**a**) MI effect magnetic field dependence protein A detection based on different Dynabeads concentration. (**b**) MI effect frequency dependence for the same protein detection in (**a**). (**c**) MI effect magnetic field dependence based on different size and concentration of Dynabeads, (**d**). MI effect frequency dependence for the same Dynabeads in (**c**). Figures (**a**) and (**b**) reproduced from [112] with permission from Springer Nature, Figures (**c**) and (**d**) reproduced from [113] with permission from John Wiley and Sons.

Wang et al. detected Dynabeads at concentrations of 0.1–100 μg/mL and claimed that the MI effect was not proportional to the Dynabead concentration [112]. As shown in Figure 7a,b, the lowest concentration (0.1 μg/mL) displayed the highest MI effect. This is because the magnetic beads were dispersed on the sensing element, so a cluster of beads was not created on the surface. If a cluster of magnetic beads forms, the adjacent magnetic fields produced by the beads would cancel each other out, resulting in a lower magnetic signal. A similar finding is shown in Figure 7c,d, where the lowest Dynabead concentration (0.1 μg/mL) shows the highest MI effect [113]. They also observed that when Dynabeads were unevenly distributed at concentrations of 1 μg/mL the MI was reduced. This reduction in MI is assumed to be due to non-uniform distribution and high-level clusters beads [113]. Comparable findings have been reported, where large MI responses have been achieved at low concentrations [111,114,115]. Rife et al. stated that the opposite dipole fields produced by the bead clusters reduces the applied external magnetic field where the resultant MR signals become lower in the presence of beads agglomeration (above 1000 beads) [34]. In this work they evaluated the stray magnetic field produced by beads (2.8 µm) at different concentrations. The magnetic field dropped by 9% when the bead distance was 2.8 μm (1156 beads) and by 23% when the bead distance was 1 μm (2530 beads). These findings concord with the results reported by Martins et al. where the MR effect dropped after a threshold of bead concentration was exceeded [116].

High-concentration magnetic bead detection is problematic and magnetic beads must be uniformly distributed on the sensing element to prevent cluster formation to avoid the offset effect induced by the stray magnetic field. To adjust the offset effect of the stray magnetic field, a perpendicular external magnetic field must be applied to create an ordered bead self-assembly process and disperse them evenly on the sensing element [117]. Therefore, a perpendicular external magnetic field is more effective than a longitudinal one for high-concentration bead detection. Alternatively, having a large sensing area is useful for preventing bead accumulation and ensuring an adequate distance between beads; the gap should be at least the bead diameter. For instance, if the sensing area of the biosensor is 3 × 3 mm^2^, the number of 1 μm beads size should not exceed 3000 × 3000. Therefore, to avoid the offset effect of the stary magnetic field, an inter-bead gap equivalent to at least the bead diameter is required, resulting in roughly 1500 × 3000 beads. 

### 5.2. Effect of The Stray Magnetic Field of Magnetic Particles on MI Response

The effect of the stray magnetic field on MI effect due to magnetic particles is subjected to on-going investigations seeking to clearly understand the underlying physics and disentangle the complex relationships. Kurlyandskaya et al. reported that the enhanced MI effect is a consequence of; firstly, the stray magnetic field of the magnetic particles disturbs the uniform externally-applied magnetic field, and secondly, the stray magnetic field of the magnetic particles alters the superposition of the magnetic field and AC, and thus changing the MI effect [107]. Phan et al. reported an increase in the MI effect because the magnetic beads were magnetically excited in the transverse direction [109]. Moreover, Wang et al. suggested that the improved MI effect can be understood as a result of; magnetic Dynabeads becoming pinned on the magnetic domain of the sensor’s sensing element [49]. The Dynabeads’ pinning field may contribute to suppressing the domain-wall movement but improving the magnetic moment cycle. Accordingly, the magnetic material’s transverse permeability is significantly enhanced because the stray magnetic field modifies the rotational magnetic permeability. In other words, the enhanced MI effect at high frequency was mainly caused by the magnetic moment rotation [118]. With regards to reduced MI effect, Wang et al. observed a drop in the MI effect caused by the longitudinal stray magnetic field resulting in field inhomogeneity, thereby diminishing the permeability and consequently the MI effect [91]. With regards to the offset MI effect, it has not been yet theoretically explained [102,106]. 

It is worth noting that, highly sensitive biosensor could be produced based on factors include, material, geometry, fabrication structure, magnetic beads, measuring circuit and magnetic field. Table 3 summarizes the optimization parameters for each factor that is required to produce a highly sensitive MI biosensor. 

## 6. Conclusions 

We have reviewed studies investigating the materials and geometries that maximize the MI performance and the measurement techniques for their characterization. The evidence suggests that the use of thick magnetic materials when fabricating MI biosensors causes a low permeability resulting in a high MI effect. Sandwich layout MI biosensors exhibit higher sensitivity than single layer sensors where no flux leakage is detected in closed-flux compared to open-flux configurations. Meander line pattern is a desirable shape to provide large MI response. MI biosensors fabricated using magnetic materials, such as amorphous ribbons, wires, or Permalloys, yield high magneto-inductive effect when the outer magnetic layer is less than the critical thickness. The MI performance is influenced by the magnetic domain configuration and it should be defined during deposition to achieve high sensitivity. However, reducing the lateral size of the sensor affects the induced domain and increases the flux leakage in open-flux configurations. Fabrication parameters, sensor size, and thickness all significantly affect MI performance and must be optimized to attain the maximum MI effect. 

The impedance value strongly depends on the measuring circuits, due to their external inductance, and they should be minimized, to attain the ideal MI effect. Calibration does not completely eliminate the external inductance in the measuring systems. However, a specialized technique to extract the intrinsic impedance of the sample can be used to produce much larger MI responses. The MI response has real (in phase) and imaginary (90 degrees out of phase) impedance values. To maximize the imaginary impedance values, the external inductance must be reduced. To maximize the MI effect as a function of frequency real and imaginary impedance parts must be kept approximately equal. 

Variations in the MI effect, including enhancement, reduction, and offset, are observed in the presence of magnetic particles but not in bead-free detection. A great increase in the MI effect is observed when there was an insulating layer between the magnetic materials and the conductive layer, or with a protective layer on the sensing element. However, the physical mechanisms of these phenomena remain unclear. 

High-concentration magnetic particles induce high-density agglomerations and clusters of particles. Particle aggregations and clusters cancel out exciting fields. This makes MI biosensors not ideal for use in quantitative assessment. Three main issues need to be addressed to detect high-concentration magnetic particles: First, the particles need to be spread out after they are dropped onto the sensor sensing element. Second, the sensor’s sensing element area must be large enough to load and accommodate all the magnetic particles so that high-density particle accumulation can be prevented. Inter-particle gaps of at least one particle diameter should be left to prevent agglomeration. Third, a perpendicular magnetic field should be employed that can uniformly distribute the magnetic particles on a flat surface. Resolving these issues would increase sensor sensitivity and make the MI effect suitable for biosensing applications.

Magnetic beads should be magnetized in the transverse direction to limit the effect of the stray magnetic field and increase the transverse permeability. Shifting of the MI curve is caused by the presence of the stray magnetic field, which must be controlled to prevent this. An accurate theoretical model needs to be constructed in future work to understand and control stray magnetic fields and prevent MI shifting. More confirmative experiments are needed, such as magnetic domain and magnetic moment observations. Moreover, further research is needed that uses experiments to demonstrate magnetic domain alignment when the protective layer is coated or is placed between the magnetic particles and the sensing element.

## Figures and Tables

**Table 1:** The highest MI effect based on geometry, materials, and structure.

MI Ratio	Material	Shape/Geometry	Structure	Ref
600%	Amorphous wires and glass-coated microwires	Wire-circular	Single layer	[50]
800%	FeCoNi magnetic tubes	Wire-circular	Sandwitch	[51]
700%	CoSiB/Si0_2_/Cu/SiO_2_/CoSiB Co-based amorphous ribbon	Rectangular single line	Sandwich	[52]
8%	NiFe/Co/NiFe Permalloy	Ellipsoid single line	Sandwich	[53]
440%	CoSi-B/Ag/Co-Si-B	Rectangular single line	Sandwich	[54]
35%	F/SiO_2_/Ti/Cu/Ti/SiO_2_/F Ferromagnetic alloy	Rectangular single line	Sandwich	[55]
120%	FeNi/Ti/Cu/Ti/FeNi Permalloy	Elongated strips	Sandwich	[56]
60%	NiFe/SIO/sub 2/Permalloy	Strip pattern	Sandwich	[57]
5.4%	Amorphous FeSiB	N/A*	Single layer	[58]
100%	Ferromagnetic CoNbZr	Rectangular double line	Sandwich	[59]
220%	Nanocrystalline FeCuNbSiB	Rectangular single line	Tri-layers	[60]
190%	NiFe/Cu/NiFe Permalloy	Meander line	Sandwich	[61]
183%	NiFe/Cu/NiFe Permalloy	Meander line	Sandwich	[62]
46%	Amorphous Co_85_Zr_3_Nb_12_	Ellipsoid	Single layer	[63]

N/A*: Not available, not provided in the reference.

**Table 2:** Highest MI effect based on magnetic particles.

MI Ratio	Material	Particle Type	Ref
350%	Co_67_Fe_4_Mo_1.5_Si_16.5_B_11_ amorphous, ribbon	Ferrofluid	[102]
125%	[Fe_19_Ni_81_]/Cu]_4_ Permalloy	Ferrofluid- iron oxide micro and nanoparticles	[103]
440%	CuBe/Fe_20_Co_6_Ni_74_ electroplated wires	Ferrofluid	[104]
417%	CoFeSiB amorphous wire	Ferrofluid	[105]
110%	(NiFe/Cu/NiFe) Permalloy	Dynabeads M-480	[106]
375%	CoFeMoSiB amorphous ribbon	Dynabeads M-450	[107]
80%	Amorphous ribbon	Dynabead M-280	[108]
70%	Amorphous ribbon	Dynabead M-100	[108]
35%	Amorphous ribbon	Nanomag-D beads	[109]
155%	FeNi/Ti magnetic materials	Ferrogels- γ-Fe_2_O_3_ magnetic nanoparticles	[110]
42%	Amorphous ribbon	Fe_3_O_4_, nanoparticles	[111]

**Table 3:** Parameters required to produce highly sensitive MI biosensor.

Material	Geometry	Fabrication	Measuring Circuit	Magnetic Particles	Magnetic Field
Amorphous alloy. Permalloy. Thick layer (<0.5 µm).	Sandwich layout. Closed path magnetic flux. Meander line pattern. Large sensing area.	Define anisotropy during deposition. Include spacer *.	Frequency of 10–100 MHz. De-embedding #. Network analyzer.	Low concentration. No agglomeration.	perpendicular magnetic field. beads should be magnetized in the transverse direction.

*, Spacer is inserted between magnetic material and conductive material. #, A specialized technique is used to obtain the impedance of the sample.
